# Autophagy, Innate Immunity and Tissue Repair in Acute Kidney Injury

**DOI:** 10.3390/ijms17050662

**Published:** 2016-05-03

**Authors:** Pu Duann, Elias A. Lianos, Jianjie Ma, Pei-Hui Lin

**Affiliations:** 1 Department of Internal Medicine—Cardiovascular Medicine, the Ohio State University, Columbus, OH 43210, USA; 2 Davis Heart and Lung Research Institute, the Ohio State University, Columbus, OH 43210, USA; jianjie.ma@osumc.edu; 3 1st Department of Critical Care Medicine & Pulmonary Services, Evangelismos Hospital, National and Kapodistrian University of Athens School of Medicine, GP Livanos and M. Simou Laboratories, Athens 10675, Greece; ealianos@yahoo.com; 4 Robert Wood Johnson Medical School, Rutgers Biomedical and Health Sciences, New Brunswick, NJ 08903-0019, USA; 5 Department of Surgery, the Ohio State University, Columbus, OH 43210, USA

**Keywords:** ischemia-reperfusion injury, nephrotoxicity, oxidative stress, kidney disease, tissue regeneration

## Abstract

Kidney is a vital organ with high energy demands to actively maintain plasma hemodynamics, electrolytes and water homeostasis. Among the nephron segments, the renal tubular epithelium is endowed with high mitochondria density for their function in active transport. Acute kidney injury (AKI) is an important clinical syndrome and a global public health issue with high mortality rate and socioeconomic burden due to lack of effective therapy. AKI results in acute cell death and necrosis of renal tubule epithelial cells accompanied with leakage of tubular fluid and inflammation. The inflammatory immune response triggered by the tubular cell death, mitochondrial damage, associative oxidative stress, and the release of many tissue damage factors have been identified as key elements driving the pathophysiology of AKI. Autophagy, the cellular mechanism that removes damaged organelles via lysosome-mediated degradation, had been proposed to be renoprotective. An in-depth understanding of the intricate interplay between autophagy and innate immune response, and their roles in AKI pathology could lead to novel therapies in AKI. This review addresses the current pathophysiology of AKI in aspects of mitochondrial dysfunction, innate immunity, and molecular mechanisms of autophagy. Recent advances in renal tissue regeneration and potential therapeutic interventions are also discussed.

## 1. Introduction

The primary function of the kidney is maintenance of body homeostasis by regulating tubular reabsorption of water, ions, glucose, nutrients and removal of waste metabolic products via glomerular filtration. The nephron (particularly proximal tubule and thick ascending limb of Henle) relies on ATP generated from renal tubular cell mitochondria to achieve sodium-coupled reclamation of 99% of filtered water. Therefore, there is high energy demand. In acute kidney injury (AKI), renal function declines rapidly and this contributes to poor patient outcomes. AKI has recently been recognized as a global healthcare issue due to associated high morbidity and mortality rates and high socioeconomic burden [1,2,3]. It is estimated that the worldwide annual occurrence of AKI reaches about 13 million people and contributes to roughly 1.7 million deaths annually [3].

AKI represents a heterogeneous disease syndrome with various causes, severity, geographical and population distributions and different outcomes. AKI is a common clinical condition and represents a diagnostic and therapeutic challenge to physicians. The disorder has a prevalence of 1%–2% among hospital admissions and 2%–7% during all hospital stays. Of these, ischemia-reperfusion injury (IRI)-related AKI is especially relevant for kidney transplantation and trauma patients in hospital admissions. In-hospital mortality rates in intensive care unit patients with AKI could reach 50%–70% [4]. In addition to high morbidity and mortality rates, AKI is associated with high cost of medical care due to lack of effective therapeutic strategies and no effective pharmacologic intervention. To put the economic burden into perspective, the estimated yearly medical expenses of AKI treatment have exceeded $10 billion in the U.S and £400–600 million in the UK [5]. However, in low- and middle-income countries, AKI mainly develops in community settings with acute endemic infections (such as malaria), toxins (venoms and poisons), and lack of available healthcare systems. Due to worldwide awareness, this information has become accessible from some population-specific (such as China) studies [6]. Despite the global attention to this common clinical condition, AKI remains a diagnostic and therapeutic challenge to clinicians. Although major advances in preclinical innovations have been made, there are “death valleys” and gaps in translating discoveries into viable therapy [7]. It is known that with timely intervention AKI is likely preventable and treatable. This has led the International Society of Nephrology to recognize such interventions as important, and develop the “0by25” initiative for AKI (zero preventable death by 2025) [3].

The most updated 2012 KDIGO (Kidney Disease: Improving Global Outcomes) clinical practice guideline has reached consensus definitions, classification and practical guidelines on AKI [8]. As an example, stage 1 AKI is defined clinically as an abrupt reduction of kidney function with concomitant decreased urinary output (<0.5 mL/kg per hour for more than 6 h) and an accumulation of serum waste products (rise of creatinine to ≥26 μmol/L within 48 h or 50%–99% rise from baseline within 7 days). Injury to the renal proximal tubular epithelium (PTE) represents the prominent cause of AKI following exposure to various renal stressors including nephrotoxins, ischemia-reperfusion injury (IRI) and sepsis. Various types of insults, intensity, dose, duration, and context, will elicit diverse forms of tubular epithelial cell death, each with distinct signaling characteristics that play distinct pathophysiological roles to the outcomes of AKI [9]. Better knowledge of epithelial cell death will enhance our understanding on pathophysiological mechanisms associated with AKI.

Autophagy is an evolutionally conserved intracellular degradation pathway responsible for maintaining cellular homeostasis. Among the various cell death pathways, autophagy is now recognized as an inducible, highly regulated process which intimately determines cell survival or death and kidney disease process. In this review, we address the current pathophysiology of AKI with emphasis on the involvement of mitochondrial dysfunction, innate immunity, and molecular mechanism of autophagy. Finally, we discuss recent advances in tissue regeneration and innovative therapeutic intervention methods.

## 2. Kidney Physiology Relevant to Acute Kidney Injury (AKI)

In humans, about 20% of cardiac output flows through roughly 3 million nephron filtration units to generate approximately 170 liters of ultrafiltrate daily. The ions, small peptides and molecules within this ultrafiltrate are reabsorbed mainly through active receptor-mediated endocytosis (transcytosis) for proteins and biomolecules. This generates roughly 1–1.5 liter of urinary excretion daily. The reabsorptive process consumes roughly 7% of normal daily energy expenditure [10]. Due to this high energy demand, renal tubule cells are rich in mitochondria. Their reabsorptive function relies heavily on normal mitochondrial oxidative phosphorylation to supply ATP as an energy source. Different nephron segments have different energy demands and, consequently, number of mitochondria; the highest mitochondria density is within the S1 segment of the proximal tubule [11]. However, in most animal models of renal ischemia/reperfusion injury (IRI) epithelial cell damage is most apparent in the S3 segment of the proximal tubule [12,13]. In this regard, the proximal tubule is exquisitely vulnerable to oxidative stress from various insults, such as sepsis, nephrotoxins, and IRI.

## 3. Animal Models of AKI

Much knowledge regarding the pathophysiology of AKI is derived from animal studies [14]. Both rats and mice have been used in animal models to study human renal physiology. However, there are notable differences among those animal models and humans. In humans, sex-related disparities exhibit little clinical relevance in the context of pharmacokinetics and pharmacodynamics related to anti-hypertensive therapies [15] and some kidney solute transporters [16]. However, due to high breeding capacity, sex-related differential distribution of specific organic anion transporters (such as Oat1 and 3) [17], and the fluctuation of hormonal conditions, female rodents usually are excluded as a suitable animal model for study of AKI. Canines are commonly used as large animal models for kidney function study because it more closely resembles human kidney function in terms of renal physiology and the practical consideration of surgical intervention in experiments. However, to meet experimental statistical significance, rodents are usually preferable models given their capacity for rapid colony expansion and easy genetic manipulation for functional studies.

The most common experimental animal AKI models are investigated with ischemia/reperfusion induced by clamping single or both renal pedicles (ischemic) followed by reperfusion injury. Chemical or nephrotoxin (such as cisplatin, folic acid and traditional herbs)-induced models, obstruction-induced injury, endotoxin sepsis models, and cecal ligation and puncture (CLP)-induced mouse sepsis models [18,19,20] have also been widely used. Recently, zebrafish has emerged as a new AKI animal model due to the great amount of offspring and because they are genomically viewed as high eukaryotic animals. Their relatively simple (only two-nephron) but well-defined kidney with representative features of mammal kidney [21] is an advantage. Moreover, nanotechnology has allowed a feasible means of measuring renal function via fluorescent nanoparticles within the tank [22]. Interestingly, nephron organoids derived from human pluripotent stem cells were recently developed to model kidney development and injury [23]. In combination with CRISPR-Cas (clustered regularly interspaced short palindromic repeat–CRISPR-associated protein) and gene-editing technologies [24,25] to generate gene-specific mutations in the kidney organoids, these innovative developments provide enormous potential to further our knowledge in molecular mechanisms of kidney disease and development [26]. That said, it has to be kept in mind that, even though these animal AKI models can reproducibly cause AKI, the human etiology of AKI is much more complex, and it is a challenge to get treatment to patients early enough in the disease course. Extensive studies are needed to accurately reproduce human AKI and translate findings from animal studies to human therapeutic interventions [14,27].

## 4. Pathophysiological Process of Acute Kidney Injury

Kidney tubular diseases are classified into two categories as either acute kidney injury (AKI) or chronic kidney disease (CKD). The devastating disease, AKI, manifested as an abrupt loss of renal function within hours to days, is especially common in hospitalized transplant and trauma patients. Multiple-etiologies such as ischemia, nephrotoxin insult, or sepsis lead to direct tubular injury [4]. Clinically, AKI results in the accumulation of plasma nitrogen metabolites (blood urea nitrogen, BUN), and serum creatinine. As for the urinary output, it is usually reduced. CKD is a condition characterized by a gradual loss of kidney function over time which could progressively lead to complications like high blood pressure, bone loss, malnutrition, and heart and blood vessel diseases. Depending on age, behavior, culture, and diet, both AKI and CKD are closely integrated and could serve as a risk factor for one another [28,29,30,31]. For example, patients surviving AKI may develop CKD or early onset of end-stage renal disease in later life due to incomplete recovery.

**Figure 1:**
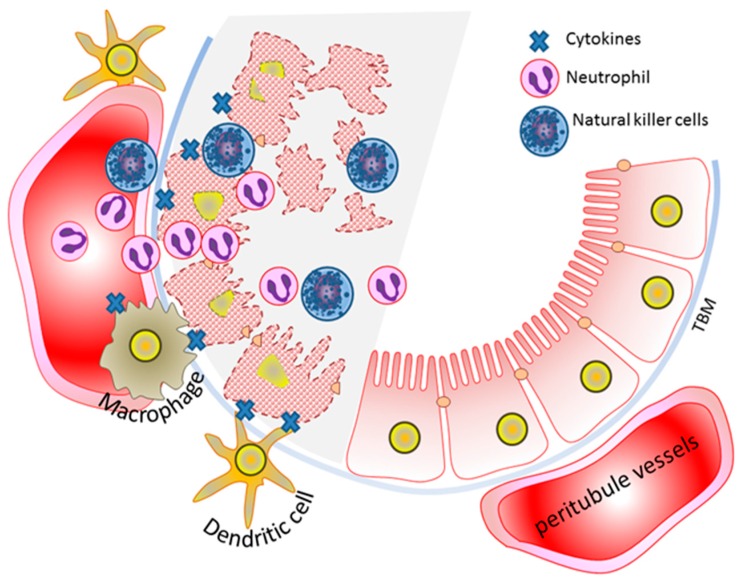
The pathophysiology of acute kidney injury involves renal tubules and vascular endothelium cell injury and inflammatory response. Diagram shows healthy tubule (**right**, unshaded) and injured tubule (**left**, shaded). The tubule cell damage involves different forms of cell death which result in loss of brush border (villi blebbing), loss of cell polarity (cytoskeletons), tubular obstruction, and cast formation. Peri-tubule vessel damage causes vascular endothelial dysfunction including: microvascular obstruction, vasoconstriction, vascular leakage, and edema. The accumulation of immune cells, such as NK cells, neutrophils, macrophages, and dendritic cells, at damaged tubules cause the release of inflammatory cytokines and further tubule cell injury. TBM, tubular basement membrane.

AKI is a complex pathophysiological response with the intricate involvement of oxidative stress accumulation, inflammatory response, tubular cell damage, and endothelial microvasculature dysfunction, all of which impact the extent of tubular cell damage [4,13] (Figure 1). Tubular cell injury usually peaks at 2–3 days post tissue damage. Upon injury, tubular epithelial cells lose cytoskeletal integrity and cell polarity, which results in mis-localization of membrane protein for ion-flux control and disruption of cell−cell communication. Tubular epithelial cell casts, derived from tubular cell debris in the lumen, are a histological characterization of tubular cell death and necrosis which commonly obstruct and reduce the urine flow.

Different modes of cell death with distinct morphologic characteristics and biochemical features are proposed to be involved in the loss of tubular epithelial cells. Of these, apoptotic cell death, multiple forms of “regulated” necrosis (also referred as necroptosis), and autophagic cell death are well documented in animal models and associated with the clinical syndrome of acute tubular necrosis and AKI [9,32]. While cell membrane integrity and morphology are largely maintained in the early events of apoptosis and autophagy, they are disrupted in necrosis. Those necrotic cells release intracellular contents including intracellular organelles, pro-immunogenic components such as Ca^2+^ ions, ATP, DNA, RNA, HMGB1 (high-mobility group protein B1), and cytokines. These released damage factors are collectively referred to as DAMPs (damage-associated molecular patterns) [33,34] and play essential roles in organ damage.

## 5. Mitochondria in Kidney Health and Disease

### 5.1. Mitochondrial Reactive Oxygen Species (ROS) Production

Mitochondria are the power house of the cells. Persistent mitochondrial dysfunction is a characteristic of AKI. In fact, loss of mitochondrial homeostasis is a key feature of tubular epithelial injury in AKI, and is characterized by mitochondrial oxidative stress and diminished cellular ATP production [35,36,37]. ATP synthesis through mitochondrial oxidative respiratory chain reaction leads to formation of oxidative stress radicals as byproducts. About 4% (or less) of total consumed oxygen is converted into superoxide radicals via electron leakage which constitutes different forms of oxidative stress [38]. Oxidative stress-causing molecules include: non-radical derivatives such as hydrogen peroxide (H_2_O_2_) and O_2_, highly reactive oxygen free radical derivatives (ROS), hydroxyl (OH-) and reactive nitrogen free radical derivatives (RNS), and peroxynitrite (ONOO-). Normally, mitochondria are equipped with powerful intrinsic antioxidant machinery to maintain intracellular redox homeostasis and keep oxidative stress under a certain threshold. Excess ROS generation is detrimental to cellular functions. It is well documented that these radicals cause modification of biomolecules, such as DNA (nuclear and mitochondrial DNAs), proteins, and lipids, as well as impair their bio-activities [36]. As such, mitochondrial dysfunction and oxidative stress accumulation play critical roles in the pathogenesis of kidney diseases [39].

### 5.2. Regulation of Mitochondria Dynamics

Mitochondria play a central role in cell survival and death signaling. It has recently been recognized that mitochondria are not only critical in energy production, but are also highly dynamic organelles with constant fusion and fission. These dynamics are essential to their size, morphology, energy biogenesis, function, and maintenance of cellular homeostasis and viability [40]. For instance, dynamic mitochondrial fusion is associated with excitation-contraction coupling in skeletal muscle [41] and maintenance of normal vital organ physiology. Impaired mitochondrial dynamics have been demonstrated in several disease states such as diabetic skeletal muscle [42], cancer cell migration [42], and neurodegenerative diseases [43].

Mitochondrial dynamics are regulated by a complex interplay between fission proteins (Drp1 and Fis1) and fusion proteins (like Mfn1, Mfn2 and OPA1) [35]. Drp1 (a large GTPase of the dynamin superfamily protein), together with its interaction with Fis1 and other adaptor proteins, undergoes post-translational modifications, like phosphorylation, ubiquitination and sumosylation, to mediate its own activation and translocation to outer mitochondrial membrane (OMM) fission sites. Drp1 n is responsible for “pinching off” the membrane stalk between two forming daughter mitochondria and returns to the cytosol upon completion of mitochondrial fission [44]. In mammalian cells, mitochondrial fusion machinery is comprised of three essential components, all of which belong to the large GTPase dynamin superfamily of proteins, including mitofusin 1 and 2 (Mfn1/2) for OMM fusion and optic atrophy 1 (OPA1) protein for inner mitochondrial membrane (IMM) fusion. Mfn1 and Mfn2 are located on the OMM and form homo- or hetero-oligomers to mediate neighboring OMM tethering and fusion. The IMM-localized OPA1 is responsible for the GTP-dependent IMM tethering and fusion and plays a key role in remodeling mitochondrial cristae during apoptosis [35,45,46]. Additionally, mitochondrial homeostasis and dynamics are also regulated by cellular physiology, including energy status, Ca^2+^ homeostasis, ROS stress, and the interaction of Bcl-2 family proteins [35,47].

During AKI, mitochondrial dynamics shift to fission, a state known to cause “mitochondria fragmentation”, which leads to subsequent cell death. It was shown that suppressed mitochondrial function and tubular damage are sustained for 6 days after IRI [48]. Moreover, it has been proposed that regulation of mitochondrial dynamics in a timely manner may prove to be an intervention strategy to prevent long-term cardiac dysfunction after myocardial infarction attack in animal model [49]. In line with this notion, suppressing mitochondrial fragmentation by mdivi-1, a chemical inhibitor against Drp-1 activity, ameliorated symptoms of IRI-induced experimental AKI [37]. Interestingly, renal cells with elevated expression of the major mitochondrial deacetylase sirtuin 3 (SIRT3), a master regulator of mitochondrial energy metabolism and oxidative stress, improved mitochondria fusion and promoted protection against AKI [50]. Together, these observations support the important role played by mitochondrial dynamics in kidney injury and repair.

Mitochondria undergo drastic morphological and functional changes during stress and injury; therefore, studying mitochondrial stress responses has been a central focus of AKI studies. Advances in live cell imaging with multiphoton microscopy allows *in vivo* high resolution of real-time visualization of subcellular structures like nuclei, endosomes, lysosomes and mitochondria [51]. In combination with different fluorescent probes, the development for organelle functional study coupled with multiphoton microscopy have provided substantial information on basic renal physiology and the underlying mechanisms of AKI. It was demonstrated that loss of mitochondrial inner membrane potential (ΔΨm) and changes in mitochondria morphology (swollen and fragmented) occur immediately (within 2 min) and are associated with a rapid (within 10 min) membrane blebbing and extensive shedding of proximal tubule apical structures, brush border, and cytosolic contents during ischemic injury, at a time when only minimal tissue damage could be observed with conventional histology techniques [51,52,53]. These studies also suggested that there is a narrow time window for application of therapeutics to prevent acute mitochondria dysfunction.

## 6. Innate Immunity and AKI

The immune system has an essential role in regulating many immune response-mediated pathologies during disease progression. This role includes the stepwise stimulation of innate immune responses with subsequent cytokine release and crosstalk between renal cells and immune cells, mainly dendritic cells and macrophages, for tissue injury resolution. It also includes adaptive immune responses with evident participation of T cells in tissue injury and repair [33].

**Figure 2:**
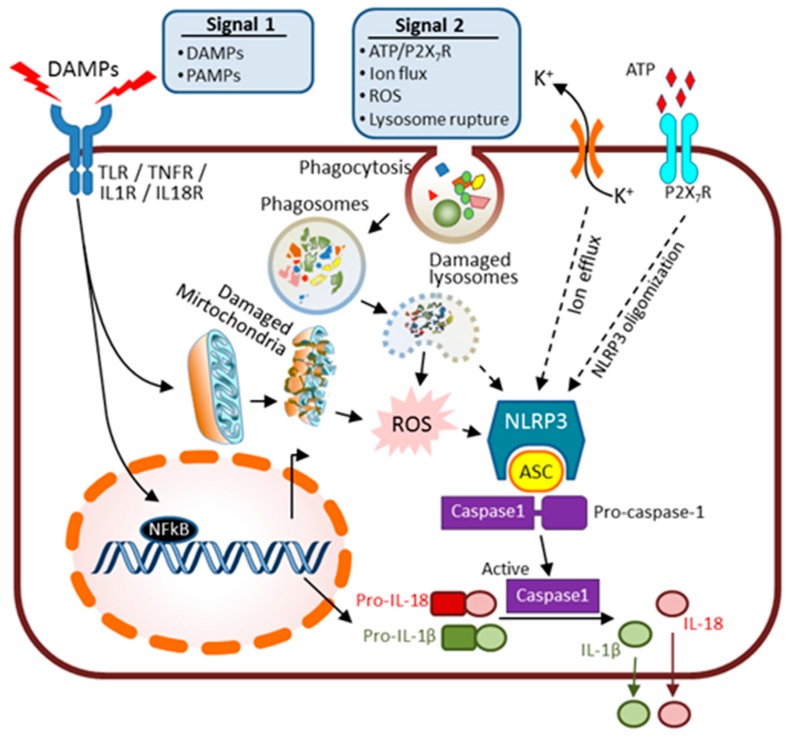
Inflammasome signaling acting in a two-step activation of IL-1β and IL-18. Activation of the NLPR3 inflammasome requires two signals. Signal 1 involves the activation of TLRs, IL-1R, IL-18R and TNFRs by DAMPs or PAMPs which then induces the transcriptional activation of NF-κB and subsequent production of pro-IL-1β and pro-IL-18; Signal 2 involves different pathways such as ion (K^+^) efflux, generation of ROS, ATP/P2X_7_R activation and lysosomal rupture/release of the endogenous cathepsins into the cytosol. Both signals coordinately induce assembly of the inflammasome complex: NLRP3 (sensor), apoptosis-associated speck-like (adaptor) and recruitment and enzymatic cleavage/activation of active caspase-1. Activated caspase-1 cleaves pre-forms of pro-IL-1β and pro-IL-18 to release the pro-immunogenic IL-1β and IL-18. Solid arrows indicate direct activation; dashed arrows indicate indirect activation.

The innate immune response can be elicited by pathogen invasion during sepsis or tissue damage factors released from injured cells (Figure 2). Pathogen invasion on the outer surface of wounded internal organs may lead to the generation of pathogen-associated molecular patterns (PAMPs) that induce sepsis if the pathogens are not controlled soon enough by local inflammatory response. Severe sepsis causes AKI. Additionally, sterile inflammation occurs frequently in many renal diseases and is triggered in response to toxins, genotoxic stresses, ischemia, or trauma. This inflammation is a response towards “damage-associated molecular patterns” (DAMPs) released from injured necrotic cells. DAMPs include: histones, HMGB1, U1snRNP, DNA/RNA from nucleus, ATP, mtDNA (mitochondrial DNA), heat shock proteins (HSPs), S100 proteins, uric acid, RNA from cytosol, and lysosomal enzymes from damaged lysosomes [33,34]. In both PAMPs and DAMPs cases, oxidative stress activates host innate defense mechanism which induces renal tubular epithelial cell necrosis while infiltrating immune cells secrete many pro-inflammatory cytokines and chemokines.

Two signals are required for innate immune response activation. As illustrated in Figure 2, inflammatory cytokines along with some DAMPs or invading PAMPs are recognized by plasma membrane-bound Toll-like receptors (TLRs) and serve as the “prime” signal (Signal 1). The TLRs’ membrane receptors serve as pattern-recognition receptors (PRRs) for the signals of DAMPs and PAMPs, and offer crucial host specificity during the early host defense phase [54]. Both TLR 2 and 4 have been shown to be upregulated in renal epithelial cells upon IRI and are crucial in initiating influx of various immune cells, such as polymorphonuclear leukocytes, lymphocytes, dendritic cells, and macrophages, into damaged interstitium (a pro-inflammatory phase) [55,56]. This step also activates the nuclear transcriptional factor NFκB to induce expression of premature forms of pro-inflammatory factors such as pro-interleukin (IL)-18 and pro-IL-1β. The introduction of a second signal (Signal 2) leads to stepwise activation and formation of an intracellular multi-protein complex, the “inflammasome” amplification loop. Several processes have been identified as the secondary signals to activate inflammasomes such as reactive oxygen species (ROS) from damaged organelles (mitochondria) [57], K^+^ ion flux [58], ATP/P2X7R [59,60] and lysosomal rupture [61,62]. The inflammasome complex further senses the danger signals outside or within cells and enhances the secretion of pro-inflammatory cytokines.

In a simplified structural model, the inflammasome-complex consists of three main components: a sensor, an adaptor, and a pro-inflammatory caspase [63] (Figure 2). The sensor proteins are mainly comprised of the intracellular NOD-like receptor, also known as “nucleotide-binding domain and leucine-rich repeat (LRR) containing receptor” family proteins (NLRPs), with NLRP3 being the best-characterized member. NLRP3 inflammasome is widely expressed in immune cells and to a lesser extent in tubular cells [64] and podoytes [57]. Activation of the inflammasome leads to overexpression and oligomerization of NLRP3, recruitment of the adaptor “apoptosis-associated speck-like (ASC)” protein and procaspase-1 enzyme. This process accounts for the final activation of caspase 1, and the processing and secretion of cytokine mediators in their mature forms including IL-1β, IL-18 and IL-33 [65].

The concept of renal inflammasome activation and its role in kidney disease has been the recent research focus in kidney injury and regeneration [34,54,66,67,68]. Interestingly, during the recovery phase of AKI, in response to microenvironmental change, macrophages are polarized to adopt a trophic phenotype and become anti-inflammatory (or pro-regeneratory). This macrophage-relevant repair process would determine the progression or resolution of tissue inflammation and subsequent tissue fibrosis [69].

## 7. Kidney Repair and Regeneration after AKI

Mammalian kidney tubular epithelium uniquely possesses a great capacity to repair and regenerate in order to restore normal epithelial integrity after AKI [12,70]. Under normal conditions, human proximal tubular cells have a slow turnover rate to maintain tissue homeostasis; however, this rate is switched to a fast proliferation mode after injury. There were strong debates about the origin of cells that initiate the repair program attempting to replenish the loss of cells from insult-induced apoptosis and necrosis [12,71]. Recent results support evidence that the terminally differentiated epithelial cells dedifferentiate upon injury, migrate along the basement membrane, then regain apparent stem-cell characteristics, proliferate to restore cell number, and expand in size to repair [71]. As mentioned above, it appears that effective kidney regeneration requires macrophage-mediated resolution of inflammation to support regeneration [69]. Furthermore, circulating bone-marrow-derived stromal cells (BMSCs) can facilitate the repair process through a microvesicle-mediated paracrine effect that transfers proteins, receptors, mRNA, microRNAs and organelles [72]. Various preconditioning interventions also support the concept of microvesicle-mediated inter-organ crosstalk playing important roles in tissue repair process [72,73].

Fibrosis is the final common characteristic of CKD which could be derived from maladaptive wound repair following AKI [13,30,74]. Several pro-inflammatory and profibrotic factors are released by injured epithelium and immune cells, which contributes to resident fibroblasts and myofibroblast activation, progressive accumulation of interstitial matrix proteins, irreversible scarring, and gradual loss of functional nephrons [75,76,77]. A better understanding of the repair/regeneration mechanism will facilitate therapeutic interventions toward AKI and help the development of pharmacological therapeutics to halt the progression of CKD.

## 8. Autophagy

**Figure 3:**
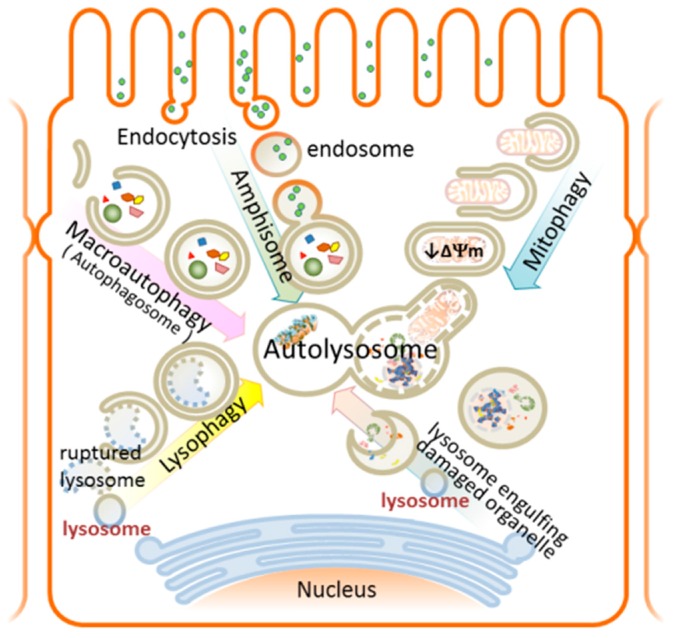
Autophagy contributes to protein degradation and damaged organelle removal in renal tubule cells. Active endocytosis occurs in the renal tubule cells for kidney ultrafiltrate reabsorption. Amphisome, a form of autophagic vacuole, is formed by fusion of an endosome with an autophagosome. Macroautophagy is triggered by the activation of an autophagic protein complex that induces LC3 recruitment to the nascent autophagosome (isolation membrane). Proteins that are committed for degradation are labeled by polyubiquitin chains and delivered to the autophagosome by the p62 scaffold protein. Fusion of autophagosomes and lysosomes, known as autolysosomes, results in the degradation of the contents. Selective autophagy like mitophagy and lysophagy are used to remove damaged mitochondria (depolarization of inner mitochondrial membrane) or ruptured lysosome, respectively, during tubule cell injury.

Autophagy (from the Greek “auto” (self) and “phagy”, meaning eating) is an intracellular degradation process utilized by eukaryotic cells as a basal “quality-control” mechanism to degrade and turnover aged or damaged cellular components in order to maintain homeostasis. As such, autophagy is critical to a wide variety of physiological and pathophysiological processes [10,78]. Basal autophagy plays important roles during development and differentiation. Additionally, autophagy is also a defense mechanism employed against environmental stress such as nutrient deprivation, aging, pathogen invasion and various disease states [79,80]. During nutrient starvation, non-selective autophagy (“macroautophagy” or “autophagy”) is applied to sequester and eliminate cytoplasmic burdens, *i.e.*, damaged organelles and protein aggregates, for subsequent use in amino acid recycling, ATP generation and anabolic protein synthesis. In certain stressed situations, selective autophagy occurs in order to remove toxic materials within cells. Specific examples include: damaged mitochondria (mitophagy), infectious bacterial particles (xenophagy), aggregating proteins (aggrephagy), or ruptured lysosomes (lysophagy) (Figure 3).

In simplified terms, autophagy is a lysosome-dependent “self-eating” process which involves multiple steps of a well-conserved sophisticated degradation process to ensure cellular homeostasis. Autophagy is a highly dynamic and context-dependent process. A set of evolutionally conserved genes and proteins, autophagy-related genes (*atg*) and proteins, originally identified in yeast, have been identified to participate in autophagy [81,82]. During autophagy, double membrane-bound vesicles form to engulf the damaged cytoplasmic constituents to generate autophagosomes. Autophagosomes then mature and fuse with lysosomes to form autolysosomes which allow cargo degradation and subsequent macromolecule recycling and regeneration. The final step of autophagy involves reactivation of mTOR (mammalian target of rapamycin), a pivotal modulator of cell growth, survival and metabolism, and the autophagic lysosome reformation.

Notably, the term “autophagy flux” is used to represent the entire dynamic autophagic process, including autophagosome formation, maturation, autophagosome-lysosome fusion, macromolecule digestion and lysosome recycling. It is of extreme importance to accurately assess autophagic flux *in vivo* and *in vitro* to fully understand the real function of autophagy in live cells, animal, and patients. Most assays use the autophagy marker protein microtubule-associated protein1 light chain 3 (LC3), a yeast ATG8 protein homologue, as readout for autophagic activity. LC3 is a ubiquitin-like adaptor protein required for autophagosome formation [83]. The cytosolic “deletion form” (LC3-I form) becomes phosphatidylethanolamine-lipidated (LC3-II form) and tightly associated with the autophagosomal membranes as an indication of autophagosome formation. Although LC3-II content is a good indicator of the autophagic vesicle number, LC3-II itself is degraded within lysosome after autolysosome formation and complicates autophagic measures. Therefore, care must be taken when interpreting the result of autophagy flux analysis [84,85,86].

## 9. Autophagy and AKI—Overview

Accumulating autophagy research corroborated the essential roles of this highly context-dependent cellular homeostasis pathway in regulating cell viability during tissue injury and repair. Balanced autophagy is critical in maintaining cellular homeostasis and viability, for damage occurs in the event of either “too much” or “insufficient” autophagy. In support of this, autophagy dysfunction has been linked to many diseases including skeletal muscle diseases, cancers, neurodegenerative diseases, systemic lupus erythematosus autoimmune disease, and others [87]. Besides mitochondrial fragmentation, severe oxidative stress (ROS) is induced during renal injury and has been implicated as an upstream signal to induce autophagy [88]. Recently, cumulative evidences support a cyto-protective role of autophagy in AKI. Several genetically modified animal models, with either tubule epithelial cell-specific or systemic deficiency of genes involved in the autophagic pathway such as *atg5*, *atg7*, LC3 knockout, beclin1 heterozygous mutant mice or the transgenic animal expressing GFP-LC3-RFP autophagic flux reporter have provided valuable tools to gain insights into how autophagy is involved in various kidney diseases and AKI [89].

Autophagosome was first identified in murine renal IRI model on tubular epithelial cells and also in human kidney transplants [90]. Later, autophagy was shown to be rapidly induced in ischemic AKI model, and autophagic flux was increased during the reperfusion phase following ischemic injury, which occurred well ahead of tissue damage [91]. Moreover, studies from transgenic animal expressing GFP-LC3 confirmed a time-dependent autophagosome (GFP-LC3 punctates) accumulation in the proximal tubule in a cisplatin–induced AKI model. In this study, apparent autophagy was induced within 6 h of treatment and peaked at day 3 in proximal tubules [92]. Tubule-specific ATG5- or ATG7- knockout mice demonstrated exacerbated IRI or cisplatin-induced kidney damage and apoptosis [92,93,94,95]. Mechanistically, in these tissue-specific autophagy-deficient AKI models, animals displayed exacerbated mitochondrial dysfunction including morphological changes, increase in ROS production, DNA damage, apoptosis with reduction in cell viability, and loss of renal function. In line with this finding, mouse models with tubule-specific deletion of Rictor/mTORC2 [96] or mTORC1 [97] demonstrated the fundamental roles of mTOR complexes in response to nephrotoxin or ischemic stresses. Consistently, autophagy is also documented in response to CLP septic AKI animal model and played a renoprotective function in proximal tubular cells [98]. Research with LPS-induced endotoxemia in septic AKI model further indicated the critical role played by mTOR in modulating autophagy, calcium signaling, and immune response [99,100]. In summary, these findings corroborate the renoprotective and pro-survival effect of autophagy in various types of AKI etiologies and highlight the importance to delineate the underlying mechanism.

It appears that efficient autophagic flux is critical to promote cell survival. Recently, an autophagic flux study using GFP-LC3-RFP transgenic mouse in ischemic AKI distinguished two populations of cells: early autophagic vacuoles (yellow signal derived from combined GFP-LC3 and RFP-LC3 punctates) and autolysosomes (red signal from RFP-LC3 punctates only). This study provided a detailed time-course of autophagy in relation to cell proliferation [89]. The results support the notion that autophagy was initiated at day 1 and autophagosome clearance occurred during renal recovery at day 3. Notably, autophagic cells appeared to be less likely to divide and repair the injured tubule cells.

Autophagy has recently been proposed to play a “dual role”—both renoprotective and detrimental, depending on the IRI experimental procedures of ischemia and reperfusion insults [101,102]. For renoprotective autophagy to happen, the ischemic duration should be limited to a threshold of 45 min for mice and 60 min for rats [101]. Within this condition, autophagosomes sequester damaged organelles like mitochondria, ER, and ribosomes and prevent subsequent ROS release and cell death. Paradoxically, detrimental autophagic responses were also reported when extended ischemic conditions or other modulations affecting autophagy were applied. Therefore, it is suggested that excessive autophagy during tissue damage causes detrimental degradation of damaged organelles and may trigger further tissue injury and cell death pathway [101,102]. Taken together, autophagy is a time- and context-dependent process, subject to dynamic fluxregulation to switch the balance between “pro-survival” or “detrimental” depending on the extent of oxidative stress. What exactly regulates this transition from protective to detrimental remains unclear. However, new tools are introduced to more accurately measure the autophagic flux in the animal models which will provide hints towards the molecular mechanisms of autophagy and its links to oxidative stresses.

### 9.1. Selective Autophagy in AKI

During AKI, large amounts of mitochondria, lysosomes and other organelles are damaged and need to be removed by selective autophagy or “organellophagy” processes [103]. To initiate selective autophagy, proteins on the membrane of damaged organelles are first recognized and ubiquitinated, followed by the recruitment of LC3 and some autophagic adaptor proteins, like p62, to the isolated membrane for autophagosome membrane formation. Eventually, the damaged organelles are incorporated into autolysosomes for degradation [104]. We will review in more detail the two types of selective autophagy—mitophagy and lysophagy in relation to AKI (Figure 3).

#### 9.1.1. Mitophagy—Mechanism and Involvement in AKI

The quality and quantity of mitochondria are under tight control in order to adjust to cellular metabolism and functional needs. Mitochondrial dysfunction represents the status of the elevation of mitochondrial calcium content, ROS production, permeabilization of outer mitochondria membrane, decrease in respiration, loss of inner membrane potential (ΔΨm), and ATP production. Notably, in animal models of AKI, mitochondrial dysfunction occurs well before renal dysfunction, as measured by increase of serum creatinine, which occurs around 12 h after IRI onset and persists for a much longer period of time than kidney function recovery [48]. Maintaining mitochondrial homeostasis is thus an important strategy in curing acute organ failure [36]. The selective removal of dysfunctional mitochondria, namely “mitophagy”, in a timely fashion via autophagy is thus critical to preserve bioenergy and prevent the release of detrimental DAMPs biomolecules and ROS. Accumulation of those damaged and aggregated biomolecules serve as strong DAMPs signals and contribute to inflammation and pathogenesis [66]. Currently, two mechanisms of mitophagy, PINK1/PARKIN-dependent pathway and Bcl2/BNIP3-dependent pathway, have been identified [105].

In the PINK/PARKIN-dependent pathway, the whole process of mitophagy is regulated by a post-translational modification circuit constituted of PTEN-induced kinase 1 (PINK1), PARKIN (an E3 ubiquitin (Ub) ligase), and mitochondria-localized deubiquitylase “Ub-specific proteases (USP)” [106,107,108]. In healthy conditions, the cytosolic level of PINK1 is kept low by serial steps of intra-mitochondrial translocation and cleavage. In damaged mitochondria, the IMM become depolarized and the PINK1 inner mitochondrial translocation is blocked which leads to its accumulation on the OMM of defective mitochondria. This is followed by PINK1-mediated phosphorylation of cytosolic PARKIN (E3 Ub ligase) and its subsequent ubiquitination. The phosphorylated and ubiquitinated PARKIN is then recruited and activated on the damaged mitochondrial surface to create many ubiquitin conjugates on mitochondria and ubiquitinate many substrates, including Mfn1 and Mfn2. The autophagy adaptor proteins LC3/Atg8 are then recruited to the complex and initiate the cascade of mitophagy to clear the damaged mitochondria. The USPs remove the Ub chain on mitochondria and counteract PARKIN-mediated Ub chain formation on cells to regulate mitochondrial homeostasis [108].

The alternative mitophagy pathway occurs to eliminate healthy mitochondria under the condition of normal reticulocyte differentiation into mature red blood cells [109]. This alternative pathway is also employed under certain hypoxia conditions as an adaptive metabolic response [110]. This process involves the direct interaction of Bcl2 and BNIP3 (Bcl2/adenovirus E1B 19 kDa protein-interacting protein 3) family proteins to prevent ROS accumulation and cell death [111]. Studies from the animal ischemic AKI models of globally or tubule-specific deletions of the apoptotic proteins Bax or Bak have confirmed their involvement as regulators of mitochondrial integrity and their roles in renal pathophysiology [112]. This also highlights the close interaction of apoptosis, autophagy (mitophagy) and kidney injury. For future therapies, it will be useful to design a targeted approach to regulate mitophagy specifically during kidney injuries for renoprotection.

#### 9.1.2. Lysophagy—Mechanism and Involvement in AKI

Lysosomes are intracellular membrane-bound acidic (about pH 4.5) organelles containing more than 50 kinds of hydrolytic enzymes known as “cathepsins” capable of degrading biomolecules such as proteins, nucleic acids, lipids and cell debris. During lysosome rupture, the lysosomes release large amounts of cathepsins, proteins, and calcium into cytosol, which result in apoptotic cell death, caspase activation and cellular dysfunction. Evidence demonstrated that damaged lysosomes are selectively sequestered by macroautophagy [104,113] (Figure 3). It is known that proximal tubule-dependent receptor-mediated endocytic vesicular trafficking and lysosomal degradation (clearance) are crucial to renal protein reabsorption and clearance [114]. Indeed, reabsorption of filtered proteins is largely limited by the capacity of lysosomal degradation instead of receptor-mediated endocytosis [115]. Moreover, it has recently been shown that the numbers of lysosomes are functional or dysfunctional, remain constant within cells [113], and that ruptured lysosomes are a strong activator for NLRP3 inflammasome [61]. Thus, maintaining cellular homeostasis with lysosome-selective lysophagy becomes especially important in AKI. In line with this finding, Maejima *et al.* [113] have reported proximal tubule-specific autophagy-deficient mice demonstrating severe nephropathy under hyperuricemia-induced lysosome rupture in kidney. Interestingly, it seems there is mitochondrial and lysosomal crosstalk in the regulation of cisplatin-induced nephrotoxicity [116]. It would be of great value to study the crosstalk between mitochondria and lysosomes in the setting of animal AKI models in real time using high resolution multiphoton microscopy.

## 10. Innovative Preclinical AKI Therapy—Targeting Cell Death and Tissue Regeneration

Current AKI treatment is largely supportive in nature as there is no effective therapeutic intervention. A great deal of effort has been devoted to developing new therapies to cure AKI. This includes research to target apoptosis with caspase inhibitors (zVAD-fmk, q-VD) [117], necroinflammation [118,119], oxidative stress, regenerative stem cell therapy [27,120,121], microvesicle-delivered paracrine effects [122,123], mitochondrial dynamics [124,125] and autophagy [126]. These studies mainly remain at the animal model and cell therapy levels but do offer innovative opportunities for translation into clinical research. Currently, several clinical trials are in progress, aiming to translate preclinical experimental research into clinical therapies (clinicaltrials.gov) [7,127].

## 11. Concluding Remarks and Future Perspectives

Presently, acute kidney injury (AKI) continues to have high morbidity and mortality with a high medical and financial burden globally. The pathophysiology of this disease is rather complex and involves multiple cell systems including renal tubules, immune cells and vascular endothelial cells. Moreover, there are intricate crosstalks among different cell death pathways, such as apoptosis, autophagy and programmed necrosis. We still need to better understand the molecular mechanisms, and identify the key players in these pathways in order to develop new therapeutic designs. However, new tools and microscopic advances are introduced to more accurately measure autophagic flux in animal models, thus providing hints toward the molecular regulation of the autophagic pathway and its links to oxidative stresses during tissue injury and repair. Several modulators have been discovered recently and offer a better understanding of this complex signaling network. Considerable progress has been made in the fields of basic immune mechanisms and kidney pathophysiology. Clinically, a web-based tool was developed for risk calculation to better predict post-kidney transplantation or “delayed graft failure”-associated AKI [128,129] and fits well into the global “0by25” initiative for AKI (zero preventable deaths by 2025) [3]. All these efforts hold great promise for both the identification of novel therapeutic targets and biomarker-based evaluation of the damage-repair process to be better translated into clinical therapies for new drug design. Perhaps strategies with a combinatorial modulation of multiple drug targets would be beneficial to the disease outcome and provide new hope for the cure of AKI and related renal diseases.

## Abbreviations

AKI	Acute kidney injury
CKD	Chronic kidney disease
IRI	Ischemic reperfusion injury
PTE	Proximal tubule epithelium
ROS	Reactive oxygen species
CLP	Cecal ligation and puncture model of sepsis
DAMPs	Damage-associated molecular patterns
PAMPs	Pathogen-associated molecular patterns
TLR	Toll-like receptors
OMM	Outer mitochondrial membrane
IMM	Inner mitochondrial membrane
